# The co‐transcriptome of uropathogenic *E* *scherichia coli*‐infected mouse macrophages reveals new insights into host–pathogen interactions

**DOI:** 10.1111/cmi.12397

**Published:** 2015-01-24

**Authors:** Charalampos (Harris) Mavromatis, Nilesh J. Bokil, Makrina Totsika, Asha Kakkanat, Kolja Schaale, Carlo V. Cannistraci, Taewoo Ryu, Scott A. Beatson, Glen C. Ulett, Mark A. Schembri, Matthew J. Sweet, Timothy Ravasi

**Affiliations:** ^1^ Division of Biological and Environmental Sciences and Engineering, Division of Computer, Electrical and Mathematical Sciences and EngineeringKing Abdullah University of Science and TechnologyThuwalKingdom of Saudi Arabia; ^2^ Division of Medical Genetics, Department of MedicineUniversity of California, San Diego9500 Gilman DriveLa JollaCAUSA; ^3^ Institute for Molecular BioscienceThe University of QueenslandSt LuciaQueenslandAustralia; ^4^ Australian Infectious Diseases Research CentreThe University of QueenslandSt LuciaQueenslandAustralia; ^5^ School of Chemistry and Molecular BiosciencesThe University of QueenslandSt LuciaQueenslandAustralia; ^6^ Institute of Health and Biomedical Innovation (IHBI), School of Biomedical SciencesQueensland University of Technology (QUT)60 Musk Ave/cnr. Blamey StKelvin GroveQueensland4059Australia; ^7^ Biomedical Cybernetics Group, Biotechnology Center (BIOTEC)Technische Universität DresdenTatzberg 47/49Dresden01307Germany; ^8^ Griffith Health Institute and School of Medical Science, Griffith Health Centre, Gold Coast CampusGriffith UniversitySouthportQueensland4222Australia

## Abstract

Urinary tract infections (UTI) are among the most common infections in humans. Uropathogenic *E* *scherichia coli* (UPEC) can invade and replicate within bladder epithelial cells, and some UPEC strains can also survive within macrophages. To understand the UPEC transcriptional programme associated with intramacrophage survival, we performed host–pathogen co‐transcriptome analyses using RNA sequencing. Mouse bone marrow‐derived macrophages (BMMs) were challenged over a 24 h time course with two UPEC reference strains that possess contrasting intramacrophage phenotypes: UTI89, which survives in BMMs, and 83972, which is killed by BMMs. Neither of these strains caused significant BMM cell death at the low multiplicity of infection that was used in this study. We developed an effective computational framework that simultaneously separated, annotated and quantified the mammalian and bacterial transcriptomes. Bone marrow‐derived macrophages responded to the two UPEC strains with a broadly similar gene expression programme. In contrast, the transcriptional responses of the UPEC strains diverged markedly from each other. We identified UTI89 genes up‐regulated at 24 h post‐infection, and hypothesized that some may contribute to intramacrophage survival. Indeed, we showed that deletion of one such gene (pspA) significantly reduced UTI89 survival within BMMs. Our study provides a technological framework for simultaneously capturing global changes at the transcriptional level in co‐cultures, and has generated new insights into the mechanisms that UPEC use to persist within the intramacrophage environment.

## Introduction

Urinary tract infections (UTIs) represent one of the most significant community‐acquired and healthcare‐associated diseases (Foxman, 2010; Horvath *et al*., 2012). Uncomplicated UTIs result in more than 14 million medical visits and account for almost $4 billion in medical expenditure each year in the USA alone (Salvatore *et al*., 2011). Approximately 50% of women will experience a UTI at some point in their life, with almost 25% of patients experiencing a recurrence within the first 6 months following treatment of the initial UTI (Salvatore *et al*., 2011). An estimated 68% of recurrent UTIs arise from the same bacterial strain that caused the initial infection (Hunstad and Justice, 2010). Uropathogenic *Escherichia coli* (UPEC) is the most common causative agent of UTIs, being responsible for ∼ 80% of all community‐acquired infections (Foxman, 2010). In the majority of acute, uncomplicated UPEC‐mediated UTIs, single cultured isolates are diagnostic of the infection (Willner *et al*., 2014).

UPEC employ a range of virulence factors, including adhesins, toxins and iron acquisition systems to colonize the urinary tract and cause disease (Totsika *et al*., 2012; Ulett *et al*., 2013). Different UPEC strains display extensive genetic diversity owing to the presence of mobile DNA elements such as ‘pathogenicity islands’, prophages and plasmids (Hacker and Kaper, 2000; Mysorekar and Hultgren, 2006; Wiles *et al*., 2008; Hunstad and Justice, 2010; Hannan *et al*., 2012). The UPEC strains UTI89 (Mulvey *et al*., 2001) and 83972 (Lindberg *et al*., 1975c; Klemm *et al*., 2007; Zdziarski *et al*., 2010) are representative of cystitis and asymptomatic bacteriuria (ABU) isolates respectively.

Acute pyelonephritis and ABU represent the two extremes of UTI. Acute pyelonephritis is a severe, acute systemic infection caused by UPEC strains containing virulence genes clustered on pathogenicity islands (Eden *et al*., 1976; Funfstuck *et al*., 1986; Stenqvist *et al*., 1987; Orskov *et al*., 1988; Johnson, 1991; Welch *et al*., 2002). Asymptomatic bacteriuria, on the other hand, is an asymptomatic carrier state that resembles commensalism. A single *E. coli* strain may be present in ABU patients at levels of more than 10^5^ colony‐forming units (CFU) ml^−1^ for months or years without provoking a host response. Because the majority of ABU‐associated *E. coli* strains are non‐haemolytic, non‐adherent and lack hemagglutination ability, early studies suggested that this behavior reflected a lack of virulence genes (Lindberg, 1975; Lindberg *et al*., 1975a, 1975b, 1975c; Eden *et al*., 1976; Kaijser and Ahlstedt, 1977). Molecular epidemiology has shown, however, that many ABU strains carry virulence genes despite failing to express the phenotype (Plos *et al*., 1990; 1995; Mabbett *et al*., 2009).

As with all infectious agents, UPEC must overcome innate immunity, a biological system compromising both cellular mediators (e.g. neutrophils) and soluble mediators (e.g. complement proteins) that act synergistically. Several studies have investigated the role of neutrophils in UPEC‐mediated pathology (Ingersoll *et al*., 2008; Sivick and Mobley, 2010; Lau *et al*., 2012; Tourneur *et al*., 2013), whereas there is a paucity of information on the interactions between UPEC and macrophages, another key cellular component of innate immunity (Tegner *et al*., 2006). We previously demonstrated that the ability of UPEC to survive in mouse macrophages differs markedly between different strains; some strains, such as UTI89, are able to survive over a 24 h infection period, whereas others, such as 83972, are rapidly killed (Bokil *et al*., 2011). This suggests that UPEC strains like UTI89 are able to subvert macrophage antimicrobial pathways, though the mechanisms responsible are still unknown. These findings are in keeping with a larger body of literature documenting intraepithelial cell survival of some UPEC strains, both *in vitro* and *in vivo* (Hunstad and Justice, 2010).

Next‐generation sequencing technologies provide a powerful approach for studying co‐transcriptomics during infection (‘t Hoen *et al*., 2008; Hegedus *et al*., 2009; Jager *et al*., 2009; Xiang *et al*., 2010; Huang *et al*., 2012; Nie *et al*., 2012; Wang *et al*., 2012). These direct sequencing methodologies allow the measurement of millions of RNA transcripts in a sample, thus enabling identification of global differences in gene expression under different growth conditions (Morozova and Marra, 2008; Wang *et al*., 2009). RNA sequencing generates information about absolute transcript levels, avoiding many of the limitations of microarrays (‘t Hoen *et al*., 2008; Llorens *et al*., 2011). To further understand the transcriptional programmes that are simultaneously activated during host–pathogen interaction, we developed an approach for isolating total RNA from co‐cultures and analysing the simultaneous changes in expression that take place in the interacting organisms. Previous studies (Hegedus *et al*., 2009; Xiang *et al*., 2010; Xiao *et al*., 2010; Ordas *et al*., 2011) have focused on either the host or the pathogen, without revealing simultaneous co‐transcriptomic changes that occur during infection. Such methods have also relied on the isolation of species‐specific RNA, which can introduce biases in the analysis.

To gain insights into novel strategies used by UPEC to subvert macrophage antimicrobial responses, we performed a global co‐transcriptomic analysis of UPEC gene expression within murine bone marrow‐derived macrophages (BMMs). We used RNA sequencing to monitor (i) the transcriptional responses of UPEC strains UTI89 and 83972 within the intramacrophage environment across an extended time course, and (ii) differences in macrophage gene expression responses to each strain. Our comprehensive approach has generated new insights into host–pathogen interactions and the possible consequences of these interactions for disease processes.

## Results

### Analysis of digital gene expression libraries

The UPEC strains UTI89 and 83972 display contrasting intramacrophage survival phenotypes; UTI89 is able to survive in significant numbers, whereas 83972 is rapidly killed (Bokil *et al*., 2011). To examine the molecular basis for this difference, we investigated the gene expression profiles of UTI89 and 83972 in BMMs in parallel over a 24 h period. For our experimental system, we used a multiplicity of infection (MOI) of 10:1, since this MOI does not have obvious effects on BMM cell viability for either of the strains. As expected, bacterial loads of UTI89 that were recovered from BMM were substantially higher than those of 83972 (Supporting Information Fig. S1). Total RNA was harvested at 2, 4 and 24 h post‐infection (hpi) and global gene expression profiles were analysed using the Illumina Hi‐Seq 2000 Digital Gene Expression Tag Profiling Kit, a tag‐based transcriptome sequencing method. cDNA libraries were prepared from gentamicin‐treated UPEC‐BMM co‐cultures, sequenced and analysed together with bacterial and BMM control samples. The RNA‐Seq files generated for all libraries were pre‐processed by a custom java script (Supporting Information Table S1).

### Mapping RNA‐Seq reads

Alignment of sequencing reads to the respective mouse and UPEC reference genomes was performed using TopHat. Sequences were mapped against their respective *Mus musculus* or *E. coli* genomes. The resulting BAM files were further used to compute alignment statistics for all libraries employing the samtool flagstat command. These processes are summarized in Supporting Information Table S2. As expected, very few sequence reads were captured from 83972‐BMM co‐cultures at 24 hpi, consistent with the observation that these bacteria were essentially cleared by BMMs at this time point. Hence, subsequent analyses of gene expression in 83972 excluded this specific condition.

### BMM transcriptional responses to UPEC

**Figure 1:**
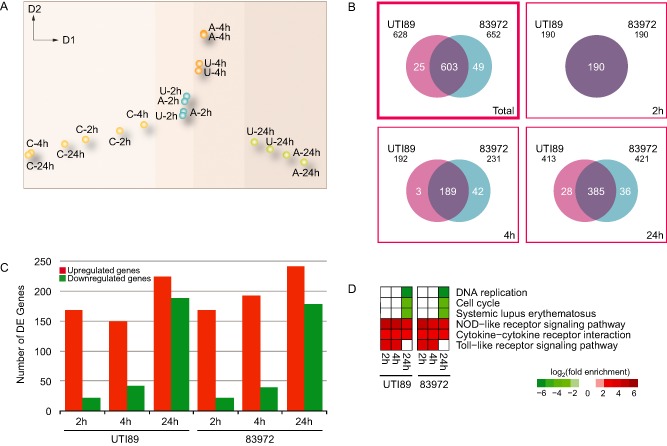
BMM transcriptome analysis. A. MCE plot of the relationships between conditions for the BMM gene sets. Each sample from two independent biological replicates is represented as a dot in a two‐dimensional space (C, control; U, UTI89; A, 83972). B. Venn diagrams quantifying the overlap in the response of BMMs to the two UPEC strains for two independent biological replicates. The numbers of DEG are shown for the total response (top left), as well as for each of the three time points (2, 4 and 24 hpi). C. Histogram of the regulation of BMM DEG showing the numbers of up‐ (red) and down‐regulated (green) genes during the 24 h infection time course. D. Pathways activated in BMMs during the course of UPEC infection. Each coloured square in the matrix represents a significant fold enrichment (log_2_) of the respective pathway term at each point. Red, up‐regulated DEG; green, down‐regulated DEG.

To explore transcriptional relationships between different conditions, we performed dimensionality reduction analysis using minimum curvilinearity embedding (MCE; Cannistraci *et al*., 2010; 2013). This analysis revealed that the BMM genes whose expression was regulated by infection were not markedly different between the two different UPEC strains at the initial stage, 2 hpi. However, after 4 hpi, a slight difference emerged, which was also maintained at 24 hpi. As expected, however, we observed that the time‐dependent regulation of gene expression (Fig. 1A) was the main pattern that emerged from the data, indicating that the macrophage transcriptional response follows a distinct temporal profile that is common to infection by both strains, and much more distinctive than the difference between the two strains.

To gain further insights into the global transcriptional changes that take place in UPEC‐infected macrophages, we catalogued differentially expressed genes (DEG) in normalized digital gene expression data through pairwise comparisons between controls (uninfected BMM cultures) and treatments (co‐cultures of UPEC‐infected BMMs) using a previously described method (Trapnell *et al*., 2012) with a threshold of a false‐discovery rate (FDR)‐adjusted *P*‐value < 0.01 in at least one of the pairwise comparisons. Using this approach, we identified 628 and 652 BMM genes that were differentially regulated following infection with UTI89 or 83972, respectively, over a 24‐h infection time course. Further analyses of the BMM transcriptome identified 603 genes that were commonly regulated by UTI89 and 83972, as well as 25 and 49 BMM genes that were differentially regulated after infection with UTI89 or 83972 respectively (Fig. 1B). The greatest divergence in responses to each strain occurred at the latest time point post‐infection.

To investigate the regulatory patterns of divergently expressed (DE) genes, we clustered genes as either up or down‐regulated. This analysis revealed a rapid response for up‐regulated genes that was maintained throughout the infection time course. In contrast, the number of down‐regulated genes was much lower at 2 and 4 hpi, but increased markedly by 24 hpi (Fig. 1C). The substantial overlap in DEG, as well as their conserved pattern of regulation, suggests that the macrophage transcriptional response to UTI89 and 83972 is broadly conserved.

### Functional annotation of DE macrophage genes

The consequences of gene expression changes associated with UPEC infection were characterized by gene ontology (GO) and pathway (KEGG) enrichment analyses of DEG using the DAVID program (da Huang *et al*., 2009a, 2009b). As shown in Supporting Information Fig. S2A, common highly enriched GO categories for up‐regulated genes included activation of inflammatory responses; regulation of chemokine, cytokine and interleukin‐6 and ‐12 production; T‐cell activation; and regulation of transcription factor (TF) activity. Common biological processes associated with down‐regulated genes included DNA replication initiation, DNA packaging, nucleosome assembly and organization, and cytokinesis. These pathways are consistent with the known activation of innate immune responses by bacterial challenge (Rosenberger and Finlay, 2003; Mogensen, 2009; Portt *et al*., 2011). As expected, the signalling pathways that were inferred, on the basis of gene expression changes, to be regulated in macrophages upon UPEC infection showed common characteristics between both UPEC strains. Signalling pathways associated with up‐regulated genes included cytokine–cytokine receptor interaction, NOD‐like receptor signalling and Toll‐like receptor (TLR) signalling pathways (Fig. 1D).

**Figure 2:**
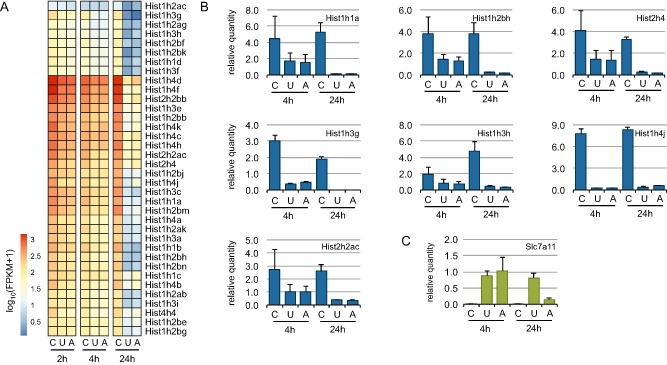
Gene regulation in BMM. A. Heat map summarizing the expression profiles of histone genes from the RNA‐Seq libraries. The values are log‐transformed FPKM counts. B. Bar plots showing the relative mRNA levels of selected histone candidate genes determined by RT‐qPCR. qPCR data represent means relative expression ± range (*n* = 2 independent experiments). C. Bar plot showing the mean relative levels of the mRNA for Slc7a11, as determined by RT‐qPCR. Error bars denote the range of the two biological replicates (C, control; U, UTI89; A, 83972).

We next independently validated both conservation and divergence in macrophage responses to UTI89 versus 83972. Many of the well‐validated TLR target genes such as cytokines (Il1a, Il1b, Il16) and chemokines (Cxcl1, Ccl8) were similarly inducible by both UTI89 and 83972 in BMM (data not shown). Although previous studies have demonstrated pathological roles for extracellular histones in mouse models of sepsis (Xu *et al*., 2009; 2011), there is little known about the regulation of histone gene expression downstream of TLR signalling. This may reflect the fact that canonical histone mRNAs are not poly‐adenylated, and so their regulated expression may not be captured by traditional microarray approaches. Interestingly, we found that a large suite of histone genes were down‐regulated in response to infection with either UPEC strain (Fig. 2A), consistent with the inhibitory effect of TLR signalling on macrophage proliferation. This observation was validated for several individual histone genes using quantitative reverse transcription‐polymerase chain reaction (RT‐qPCR; Fig. 2B). Our attempts to validate the small number of macrophage genes differentially regulated by UTI89 and 83972 (Fig. 1B) using qPCR were less successful; however, we did confirm differential regulation of the cystine/glutamate exchanger Slc7a11. Whereas *Slc7a11* mRNA expression was similarly up‐regulated by UTI89 and 83972 at 4 hpi in infected BMM, its expression remained elevated at 24 hpi in UTI89‐infected BMMs but was significantly reduced at this time point in 83972‐infected BMMs (Fig. 2C).

**Figure 3:**
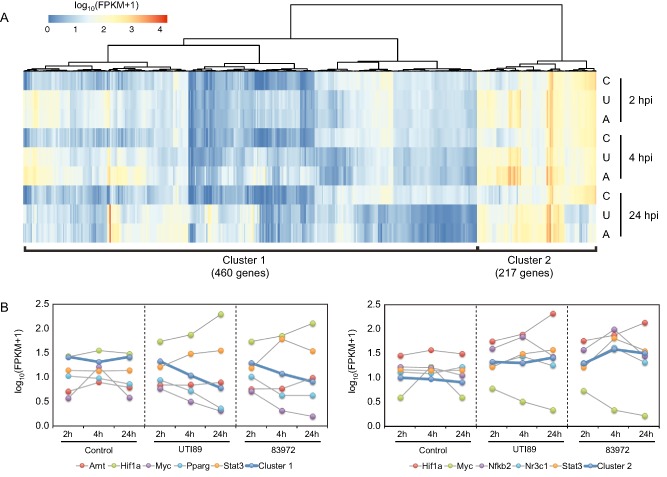
Transcription factors associated with DE BMM genes. A. Heat map showing results of k‐means clustering of BMM DEG. The values are log‐transformed FPKM counts for all DEG across all RNA‐Seq libraries in the dataset. B. Expression profiles of TFs associated with binding motifs from the TFBS analysis that are highly correlated with each cluster. Each TF is represented with a different colour, whereas the cluster mean expression is coloured blue. All values are log‐transformed FPKM counts. C, control; U, UTI89; A, 83972.

Clustering analysis separated DE macrophage genes into two major clusters (Fig. 3A). Cluster 1 (Fig. 3B), which contained 460 genes, was positively correlated with the profiles of the TFs *Arnt* (aryl hydrocarbon receptor nuclear translocator), *Myc* and *Pparg* (peroxisome proliferator‐activated receptor gamma); and negatively correlated with the profiles of *Hif1a* (hypoxia‐inducible factor 1‐alpha) and *Stat3* (signal transducer and activator of transcription 3); and enriched for pathways associated with DNA replication, cell cycle and systemic lupus erythematosus. Cluster 2 (Fig. 3B), which contained 217 genes, was positively correlated with the profiles of the TFs *Hif1a*, *Nfkb2* (nuclear factor of kappa light polypeptide gene enhancer in B cells 2), *Nr3c1* (nuclear receptor subfamily 3, group C, member 1) and *Stat3*; negatively correlated with the profile of *Myc*; and enriched for pathways associated with cytokine–cytokine receptor interaction and NOD‐like receptor, TLR, chemokine and Jak‐STAT signalling. These results are in keeping with expectations; TLR signalling activates *NF‐κB* and *HIF* (Rossol *et al*., 2011) and inactivates CSF‐1 signalling. Various studies link the biology of *CSF‐1*, *Myc* and *Pparg* (Dey *et al*., 2000; Bonfield *et al*., 2008; Pello *et al*., 2012), and as expected, down‐regulated genes have an association with *Myc* and *Pparg*. That is, TLR signalling switches off signalling via CSF‐1, which itself can signal, in part, by *Myc*; hence, TLR signalling down‐regulates *Myc* responses. Apart from the expected patterns, our analysis suggests a potential association between inactivation of CSF‐1 signalling and *Arnt* (partner for *Hif*) responses, which has not been reported previously.

### UPEC transcriptional responses upon infection of BMMs

**Figure 4:**
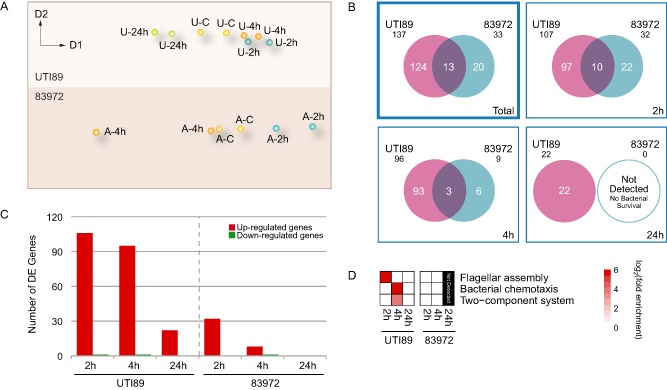
UPEC transcriptome analysis. A. MCE plot of the relationships between conditions for the UPEC gene sets. Each sample from two independent biological replicates is represented as a dot in a two‐dimensional space (C, control; U, UTI89; A, 83972). B. Venn diagrams quantifying the overlap in the response of the UPEC strains in the intramacrophage environment for two independent biological replicates. The numbers of DEG for the total response (top left), as well as for each of the three time points (2, 4 and 24 hpi) are shown. C. Histogram of the regulation of UPEC DEG showing the numbers of up‐ (red) and down‐regulated (green) genes during the 24‐h infection time. D. Pathways activated in UPEC during the course of infection. Each coloured square in the matrix represents significant fold enrichment (log_2_) of the respective pathway term at each point. Red, up‐regulated DEG; green, down‐regulated DEG.

As was the case with the macrophage gene expression analysis, we initially investigated transcriptional responses in both UPEC strains by performing dimensionality reduction analysis using MCE (Cannistraci *et al*., 2010; 2013). We identified specific differences in the regulated expression of UPEC genes within the intramacrophage environment (Fig. 4A), which likely reflects the differential pathogenicity and capacity for intramacrophage survival of both strains (Supporting Information Fig. S1; Bokil *et al*., 2011).

To gain insights into the global transcriptional changes that occur in UPEC during macrophage infection, we applied the same method described above and performed pairwise comparisons between controls (UPEC cultures alone) and treatments (co‐cultures of UPEC‐infected BMMs). Again, we employed an FDR‐adjusted *P*‐value < 0.01 in at least one of the pairwise comparisons in our dataset as the threshold for differential expression. In total, we identified 137 UTI89 genes and 33 83972 genes that were differentially regulated in BMMs. Surprisingly, an analysis of the intramacrophage transcriptomic profile identified only 13 regulated genes that were common to both strains, whereas 124 genes were uniquely regulated in UTI89, and 20 genes were uniquely regulated in 83972 (Fig. 4B). The UPEC transcriptional profiles also revealed a number of strain‐specific responses; a substantial number of UTI89 genes were up‐regulated at 2 and 4 hpi, whereas a much smaller number of 83972 genes were up‐regulated at these time points (Fig. 4C). This is consistent with the initiation of a UTI89 transcriptional programme that permits intramacrophage survival. In contrast to the transcriptional responses observed for macrophage genes, few UPEC genes were down‐regulated over the infection time course.

### Functional annotation of DE UPEC genes

The consequences of gene expression changes associated with UPEC infection were characterized by GO and pathway (KEGG) enrichment analyses of DEG using the DAVID program (da Huang *et al*., 2009a, 2009b). This again provided clear evidence of strain‐specific biological responses within the intramacrophage environment (Supporting Information Fig. S2B). Highly enriched GO categories associated with the up‐regulated transcriptional programme included genes associated with chemotaxis and motility in UTI89 and genes involved in protein folding in 83972. We did not identify any significantly enriched GO categories associated with down‐regulated genes in common for the two strains. In keeping with the above findings, an analysis of the pathways activated during UTI89 infection revealed enrichment for pathway terms associated with bacterial chemotaxis and flagellar biosynthesis (Fig. 4D). We did not observe significant enrichment for any pathway term in the case of 83972.

### UPEC genes associated with intramacrophage survival

**Figure 5:**
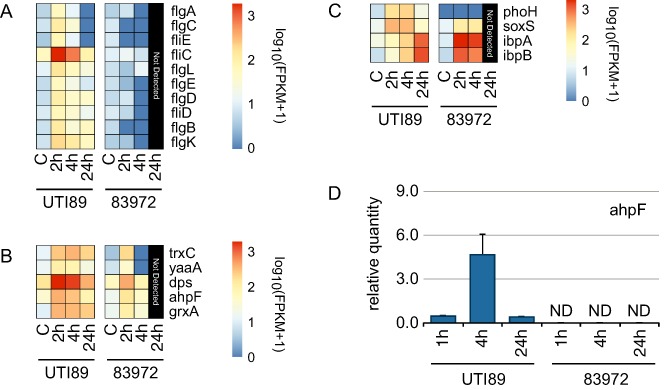
Regulation of UPEC flagella, OxyR regulon and hydrogen peroxide‐induced genes in the intramacrophage environment. A–C. Heat maps summarizing the RNA‐Seq‐derived expression profiles of flagellar genes (A), OxyR regulon genes (B) and hydrogen peroxide‐induced genes (C). All values are log‐transformed FPKM counts. D. Bar plot showing the relative mRNA levels of *ahpF*, as determined by RT‐qPCR. qPCR data represent mean relative expression ± range (*n* = 2) of two biological replicates; ND, not detected.

We hypothesized that genes selectively up‐regulated by UTI89 may contribute to intramacrophage survival. Such genes included those encoding flagella and those associated with protection against oxidative stress. Notably, several genes encoding flagella‐related proteins were uniquely regulated in UTI89; these DE flagella genes included *flgA‐F*, *flgK*, *flgL*, *fliC‐E*, *fliQ*, *motA* and *motB*. Figure 5A shows the expression patterns of all flagella‐related genes in UTI89 versus 83972, revealing strong up‐regulation at 2 h with a subsequent gradual decrease over time.

A common mechanism used by bacterial pathogens to avoid host innate immune pathways is to employ defence mechanisms against oxidative stress (Imlay, 2013). We therefore clustered the expression patterns of all the OxyR regulon genes (Fig. 5B) and the most strongly hydrogen peroxide‐induced genes in both UPEC strains (Fig. 5C). Interestingly, the OxyR regulon, which included alkyl hydroperoxide reductase subunit F (*ahpF*), *dps* (DNA starvation/stationary phase protection protein), *grxA* (glutaredoxin 1), *trxC* (thioredoxin 2) and *yaaA*, was strongly up‐regulated in UTI89, with the effect being apparent at 2 hpi and peaking at 4 hpi. Hydrogen peroxide‐inducible genes, which included *ahpF*, *dps*, *grxA*, heat shock proteins/chaperones (*ibpA*, *ibpB*), *phoH*, *soxS* (DNA‐binding transcriptional dual regulator), *trxC* and *yaaA* showed a similar pattern, with genes being significantly up‐regulated in UTI89 and less so in 83972. Differential regulation of *ahpF* in UTI89 versus 83972 within macrophages was validated by RT‐qPCR (Fig. 5D).

**Figure 6:**
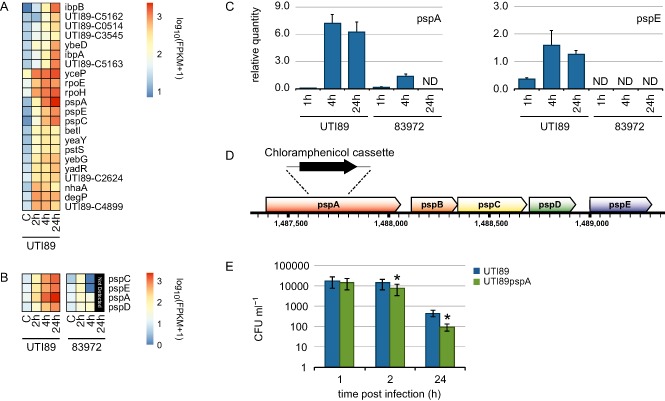
UPEC genes associated with intramacrophage survival. A and B. Heat maps summarizing the RNA‐Seq‐derived expression profiles of UTI89 genes elevated at 24 hpi (A) and UPEC Psp genes (B). C. Bar plots showing the relative quantity of pspA and pspE mRNA, as determined by RT‐qPCR. qPCR data represent mean relative expression ± range (*n* = 2) of two biological replicates; ND, not detected. D. Insertion site for creation of UTI89 *pspA* mutant. E. Intramacrophage survival of UTI89 and UTI89*pspA*. BMMs were infected at an MOI of 10 and intracellular bacterial survival was assessed at 1, 2 and 24 h of infection. Data are compiled from three independent experiments, and show mean ± standard deviation (*P < 0.05).

Although UTI89 persists at 24 h within macrophages, only a relatively small percentage of bacteria (< 5%) survive within BMM at 24 hpi compared with the bacterial loads at 2 hpi (Supporting Information Fig. S1). We therefore reasoned that changes in UPEC gene expression at 24 hpi might be linked to intramacrophage survival. We identified 22 genes that were highly up‐regulated (> 3‐fold) by UTI89 at 24 hpi (Fig. 6A). The most highly expressed of these included those encoding *ibpB* (encoding a small heat shock protein); *pspACDE* (encoding the phage‐shock protein system); *rpoE* and *rpoH* (encoding sigma factors); *smpA* (encoding outer membrane lipoprotein); *yadR* (encoding the iron‐sulphur cluster insertion protein ErpA); *yceP* (encoding the biofilm formation regulatory protein BssS); *yebG* (encoding DNA damage‐inducible protein); and UTI89_C2624 and UTI89_C5162‐3 (encoding proteins of unknown function).

Phage‐shock‐protein (Psp)‐related genes, which are required for bacterial survival during extracytoplasmic stress responses and changes in pH (Darwin, 2013), were significantly up‐regulated in UTI89 compared with 83972 (Fig. 6B). Reverse transcription‐polymerase chain reaction confirmed the elevated expression of *pspA* and *pspE* in UTI89 compared with 83972 at 24 h post‐infection (Fig. 6C). Finally, we validated the impact of *pspA* for intramacrophage survival of UTI89 by constructing a UTI89*pspA* mutant (Fig. 6D), and testing it for intramacrophage survival during a 24 h infection time course in BMM. In this assay, the UTI89*pspA* mutant was significantly reduced for intracellular survival compared with the wild‐type strain (Fig. 6E).

## Discussion

In this study, we determined the co‐transcriptomic programme of UPEC‐infected primary mouse macrophages during an infection time course. The use of two UPEC strains that differed in their ability to survive in these cells enabled the identification of both common and UPEC strain‐specific responses. To our knowledge, this is one of the first RNA‐Seq studies that have simultaneously measured the transcriptomes of both the host and pathogen during an infection (Humphrys *et al*., 2013). Using only open‐source tools, we developed a computational framework that was capable of successfully separating, annotating and quantifying the mammalian and bacterial transcriptomes. Whereas previous studies (Mysorekar *et al*., 2002; Bower *et al*., 2009; Hagan *et al*., 2010; Duell *et al*., 2012) have been limited to microarrays, the RNA‐Seq analysis reported here provides a more sensitive, comprehensive, and unbiased coverage of the entire transcriptome. We achieved an average sequencing depth of approximately 24 million tags per library and identified 677 BMM and 157 UPEC genes that were differentially expressed following UPEC infection. By mapping our RNA‐Seq tag data onto transcript databases and genomic sequences, we were able to identify genes that were regulated upon UPEC challenge.

Minimum curvilinearity embedding confirmed the distinct temporal cascade of BMM responses to UPEC as well as differences in transcriptional programmes of the two UPEC strains. The sets of DEG and regulatory processes and pathways identified in both BMMs and UPEC strains suggest coordinated expression and mutual influences. Our study detected 603 DEG in BMMs that were common to infection with UTI89 and 83972, accounting for the vast majority of changes in BMM gene expression. This was not unexpected, given that both UTI89 and 83972 present pathogen‐associated molecular patterns, such as lipopolysaccharide that is recognized by TLR4. Macrophage‐expressed genes that showed differences in regulation in response to these two strains were a very minor component of the total signature and their significance remains to be determined. Despite the general conservation of the BMM response against UPEC infection, we nonetheless expected to detect some selective BMM responses at 24 hpi for UTI89 compared with 83972, since the former condition reflects macrophages that are continuing to cope with intracellular UPEC, whereas the latter corresponds to macrophages that have cleared the infection. Consistent with this supposition, we found that at 24 hpi, BMM responses showed a greater divergence between UTI89 and 83972 (47.8% overlap) compared with earlier time points where this difference was not as evident. Of interest in this regard, was our validation of differential regulation of the cationic amino acid cysteine/glutamate antiporter Slc7a11, which is required for glutamate uptake and glutathione synthesis (Bannai, 1986; Hayes and McLellan, 1999; Pompella *et al*., 2003; Shih *et al*., 2006). This gene showed a sustained up‐regulation at 24 hpi following infection with UTI89, whereas it was only transiently up‐regulated after infection with 83972. Given that macrophage antimicrobial responses typically involve subjecting pathogens to oxidative stress, the sustained expression of Slc7a11 upon infection with UTI89 may be required to maintain glutathione levels and cytoprotection during stress responses.

Our data is consistent with previous reports showing inducible cytokine and chemokine expression, as well as activation of pro‐survival pathways (Sester *et al*., 1999; 2006), during bacterial infection and/or TLR stimulation of macrophages. Moreover, the down‐regulation of DNA replication and cell cycle genes (Fig. 1D) is consistent with the known inhibitory effects of TLR4 signalling on proliferation of cycling macrophages (Sester *et al*., 1999; 2006). We also found that the genes encoding histones H1, H2, and H4 were dramatically down‐regulated at 24 hpi, and we further confirmed the regulated expression of several of these using RT‐qPCR. A member of the H2A histone family, *Hist2h2aa1*, was also significantly down‐regulated at 24 hpi *in vivo* in mouse bladder colonized with UPEC CFT073 (Tan *et al*., 2012), which is consistent with these findings. Interestingly, previous studies have reported a pathological role for extracellular histones during LPS‐induced septic shock (Xu *et al*., 2009; Li *et al*., 2011). Xu *et al*. (2011) revealed that antibodies against extracellular histones rescued animals from LPS‐mediated death (Xu *et al*., 2011), and a previous study showed that extracellular histones mediate endothelial dysfunction, organ failure and death during sepsis (Semeraro *et al*., 2011). Our findings could thus either reflect a host attempt to reduce inflammatory responses upon cell death and histone release, or the consequence of growth‐inhibitory effects of TLR agonists on proliferating macrophages, which would also be expected to lead to down‐regulated histone expression.

A transcription factor‐binding site (TFBS) analysis of the promoter sequences of the two BMM DE gene clusters further showed significant enrichment of seven motifs associated with TFs that have key roles in macrophage functions. The cluster containing inducible inflammation‐related genes correlated positively with the expression of *Hif1a*, a key pro‐inflammatory transcription factor that drives macrophage inflammatory responses and is up‐regulated in UPEC‐infected mouse bladder at 2 hpi and 24 hpi (Duell *et al*., 2012; Tan *et al*., 2012); *Nfkb2*, which is activated by a wide variety of stimuli such as cytokines, oxidant‐free radicals and bacterial or viral products; the glucocorticoid receptor *Nr3c1*, which up‐regulates the expression of anti‐inflammatory genes and/or represses the expression of pro‐inflammatory genes; and *Stat3*, a transcriptional activator stimulated in response to cytokines and growth factors. *In vivo*, both *Nfkb2* and *Stat3* are immediately up‐regulated in the bladder following UPEC infection (Duell *et al*., 2012). On the other hand, the cluster containing down‐regulated cell cycle‐related genes correlated positively with the expression of *Arnt*, which is involved in the induction of several enzymes that participate in xenobiotic metabolism; *Myc*, which is required for cell proliferation in response to mitogenic stimuli such as CSF‐1; and *Pparg*, which regulates fatty acid storage and glucose metabolism. *Myc*, *Pparg* and *Csf1* are all differentially expressed in bladders of mice infected with UPEC (Duell *et al*., 2012) supporting the *in vivo* relevance of these findings.

In contrast to the general conserved pattern of BMM responses to both UPEC strains, the individual transcriptional responses of the two strains in BMMs differed markedly. This is consistent with their contrasting survival patterns in macrophages. Minimum curvilinearity embedding confirmed the different transcriptional programmes of the two UPEC strains, revealing 124 UTI89‐specific DEG, 20 83972‐specific DEG and 13 DEG that were commonly regulated in both pathogens upon infection of BMMs. Given the different survival patterns of both UPEC strains in macrophages, we expected to detect increased expression of genes specific to UTI89 within macrophages at 24 hpi.

Previous studies have shown that flagella contribute to the virulence of a number of pathogenic species (Tomich *et al*., 2002). Some bacteria, for example, *Salmonella enterica* Typhimurium and *Yersinia enterocolitica*, use flagella for invasion of epithelial cells (McNally *et al*., 2007; Ibarra *et al*., 2010). Uropathogenic *Escherichia coli* also employ flagella for the invasion of renal collecting duct cells (Pichon *et al*., 2009), but flagella‐enhanced uptake of UPEC by macrophages has not been investigated. Our co‐transcriptomic analysis revealed the up‐regulation of multiple genes associated with flagella biosynthesis. This could reflect inducible gene expression in the intramacrophage environment or selective uptake by macrophages of a sub‐population of flagella‐expressing UPEC, which would generate a similar gene expression profile. The acute up‐regulation at 2 hpi and subsequent down‐regulation at 24 hpi would suggest the latter may be the case.

Murine macrophages employ both rapidly produced reactive oxygen species (ROS) and reactive nitrogen species (RNS), produced in a delayed fashion, as bacterial clearance strategies (Flannagan *et al*., 2009). The observation that UPEC strain UTI89 survives in macrophages suggests that it can overcome these pathways. Although the genes that respond to ROS have been well characterized in *E. coli* K‐12, their expression during UPEC infection and their role in intramacrophage survival have not yet been studied. Our transcriptomic approach identified UPEC genes that are likely involved in defence against ROS and RNS. Disturbances in the normal redox state of cells can cause toxic effects through the production of peroxides and free radicals that damage all components of the cell, including proteins, lipids and DNA. An earlier study confirmed that the peroxide response regulator OxyR activates most of the genes that are highly induced by hydrogen peroxide (Zheng *et al*., 2001). Our results show that the response of UTI89 to oxidative stress (up‐regulated expression of *dps*, *grxA*, *ahpF*, *soxS*, *trxC*, *ibpA* and *ibpB*) is more robust than that of 83972. These differences imply that part of the UTI89 intramacrophage survival strategy includes robust protection against oxidative stress.

Several UTI89 genes were up‐regulated in BMM at 24 hpi, and we selected genes encoding the Psp system for further functional analysis. The Psp system responds to extracytoplasmic stress and contributes to the virulence of several pathogens, including *Y. enterocolitica* and *S.* Typhimurium (Karlinsey *et al*., 2010; Yamaguchi and Darwin, 2012). PspA, the master effector of the Psp system, mediates its response via a dual mechanism: (i) binding to the transcriptional regulator PspF and preventing it from activating the transcription of *pspACDE* in the absence of extracytoplasmic stress, and (ii) binding to the cytoplasmic membrane‐localized proteins PspB and PspC in the presence of extracytoplasmic stress, thus releasing PspF to induce *pspACDE* transcription (Yamaguchi *et al*., 2013). We confirmed a role for the Psp system in UPEC by constructing a *pspA* mutant (UTI89*pspA*) and demonstrating reduced intramacrophage survival of this mutant. Despite this statistically significant reduction in survival within macrophages, the biological significance of this effect is at this stage unknown. Inactivation of other stress response systems on the *pspA* mutant background may be required to reveal a more striking phenotype.

In summary, we have demonstrated the capacity to employ co‐transcriptomics to study host–pathogen interactions. Our novel approach revealed new insights into the mechanisms used by UPEC to avoid macrophage responses and persist in the intramacrophage environment, and has identified multiple target genes for further functional studies. Finally, this manuscript is one of the first to successfully evaluate the expression profiles of two organisms from the same sample using RNA sequencing, and will make an excellent resource for other studies that aim to perform similar analyses in different host–pathogen systems.

## Experimental procedures

### TIER I: *in vitro* infection assays

#### Ethics statement and animal experimentation

A University of Queensland institutional animal ethics committee approved all animal experimentations. Female C57BL/6 mice (6–8 weeks old) were purchased from the Animal Resources Center, Australia. Murine BMMs were generated by the *in vitro* differentiation of bone marrow cells from C57BL/6 mice on bacteriological plastic plates in the presence of 10 000 U ml^−1^ recombinant human CSF‐1 (a gift from Chiron) for 6 days, after which cells were harvested and replated onto tissue culture plastic in the presence of CSF‐1 for infection on day 7. BMM were maintained at 37°C (5% CO_2_) in RPMI 1640 supplemented with 2 mM L‐glutamine (GlutaMAX), 10% heat‐inactivated fetal bovine serum and 50 U ml^−1^ penicillin and 50 μg ml^−1^ streptomycin (Life Technologies).

#### Culture of bacterial strains and infection of mouse BMMs

*Escherichia coli* UTI89 is a well‐characterized cystitis isolate (Mulvey *et al*., 2001). *Escherichia coli* 83972, which was carried without symptoms by a young female, was originally isolated from the urine of this individual (Andersson *et al*., 1991; Roos *et al*., 2006). For macrophage infection assays, UPEC strains were cultured statically in Luria‐Bertani (LB) broth at 37°C overnight. Type 1 fimbriae expression was assessed by yeast cell agglutination prior to infection as previously described (Schembri *et al*., 2000). Bacterial cells were centrifuged and washed in phosphate buffered saline (PBS), and then resuspended in antibiotic‐free media at a concentration of 2 × 10^8^ CFU ml^−1^. Viable CFU counts of bacterial inocula were routinely confirmed in every infection assay by serial dilution and plating on LB agar.

Intramacrophage survival assays were performed essentially as described previously (Bokil *et al*., 2011). Briefly, following overnight adherence in antibiotic‐free media, BMMs were infected for 1 h with bacteria at an MOI of 10. Extracellular bacteria were killed by washing twice in 200 μg ml^−1^ gentamicin, followed by 1 h incubation in media containing the same gentamicin concentration. Subsequent exclusion of extracellular bacteria for the duration of the experiment was performed by incubation in 20 μg ml^−1^ gentamicin. At appropriate time points (1 h, 2 h and 24 h), cells were washed twice with antibiotic‐free media, and then lysed with PBS/0.01%Trition X‐100. Lysates were cultured on LB agar plates overnight at 37°C and colony counts were used to assess intracellular bacterial loads. For standard infection assays, complete exclusion of viable extracellular bacteria was confirmed by performing colony counts on culture supernatants.

### TIER II: RNA preparation and sequencing

#### Macrophage infection and mRNA isolation, enrichment and purification

Control macrophages, control bacteria and infected macrophages were incubated for an additional 1, 3 and 23 h at 37°C in a humidified 5% CO_2_ atmosphere, after the initial 1 h infection. Cells were then washed twice with antibiotic‐free media, and then lysed on ice for RNA isolation and purification (RNeasy; Qiagen, Germantown, MD, USA). Microbial total RNA in co‐culture samples was enriched (MICROBEnrich, Ambion). rRNA was removed from all purified RNA samples using kits targeting mammalian and gram‐negative bacterial rRNAs (Ribo‐Zero; Epicenter, Madison, WI, USA). Prior to sequencing, all samples were further quantified and examined for protein and reagent contamination using a Nanodrop ND‐1000 spectrophotometer. RNA samples for analysis were selected based on a 28S/18S rRNA band intensity of 2:1, a spectroscopic A_260_/A_280_ nm ratio of 1.8–2.0 and an A_260_/A_230_ nm ratio > 1.5.

#### RNA sequencing

Next‐generation sequencing analyses were performed for two biological replicates on an Illumina Cluster Station and the Illumina HiSeq 2000 System using primarily reagents from the Illumina Gene Expression Sample Preparation Kit and the Illumina Sequencing Chip (Flowcell; Illumina, San Diego, CA, USA). Sequence tags were prepared using the Digital Gene Expression Tag Profiling Kit (Illumina), according to the Illumina protocol.

### TIER III: data pre‐processing

Image analysis, base calling and quality calibration were performed using the Solexa Automated Pipeline. Quality control of RNA‐Seq reads were pre‐processed by a custom java script. All sequences generated have a length of 101 bases and have been submitted to the BioProject database of NCBI under BioProject ID: PRJNA256028.

### TIER IV: alignment and differential gene expression analysis

#### Alignment of reads

Bowtie indexes were created for the mouse (version 37.1) and the two *E. coli* strains (UTI89 and 83972) using the bowtie‐build algorithm and reference sequences from the GenBank database. Our protocol began with mapping of the raw RNA‐Seq reads (fastq files) to the reference genomes using TopHat. TopHat uses Bowtie as an alignment engine and breaks up reads that Bowtie cannot align on its own into smaller pieces (Kim and Salzberg, 2011). Using the standard parameters, we mapped both reads of our paired end libraries. All simulations were performed using a 30 core, high‐memory node cluster system; total computation duration was 3 h.

#### Transcript annotation

After running TopHat, the resulting alignment files were provided to Cufflinks to generate a transcriptome assembly for each condition. During this analysis step, adapter tags; mitochondrial sequences; poly A, poly C and phiX sequences; and remaining ribosomal sequences were filtered out. Estimated normalized expression levels were reported in fragments (i.e. reads) per kilobase of exon per million mapped reads (FPKM). These assemblies were compared with annotation files using the Cuffcompare utility, which is included in the Cufflinks package (Roberts *et al*., 2011; Trapnell *et al*., 2012).

#### Differential expression analysis

The reads and assemblies were imported to Cuffdiff, which calculates expression levels and tests the statistical significance of observed changes. For comparison of DEG across samples, the number of raw clean tags in each library was normalized to FPKM using the Cufflinks package. The minimum number of alignments in a locus needed to test for significance of changes in that locus between samples was set to 50 fragment alignments. If no testing was performed, changes in the locus were deemed insignificant, and the changes observed in the locus did not contribute to corrections for multiple testing. The Cuffdiff output files were then imported to cummeRbund, which plots abundance and differential expression results as commonly used expression plots for quality control (Trapnell *et al*., 2012).

### TIER V: functional analysis

#### Dimensionality reduction

Dimensionality reduction is necessary for exploring the relationships between conditions in our experiment. The MCE method performs a non‐linear dimension reduction by embedding high‐dimensional data points into a lower dimensional space using the minimum curvilinear kernel in combination with multidimensional scaling (MDS; Cannistraci *et al*., 2010) or in alternative the singular value decomposition (Cannistraci *et al*., 2013). The non‐linear data distances for MDS or SVD were computed and stored in the minimum curvilinear kernel as the traversal distances over the minimum spanning tree between the data points (in our study the samples' conditions) in the multidimensional space (in our case, the gene space). The minimum spanning tree was constructed from the Pearson correlation‐based distances between the samples (Cannistraci *et al*., 2010):

$$\begin{array}{l} correlation\_based\_distance\left(x,\;y\right)\\ \;=1-Pearson\_correlation\left(Sample_{x}\text{​},\;Sample_{y}\right) \end{array}$$

MCE is a parameter‐free projection algorithm that was shown to be particularly effective in discriminating classes in small‐*n* (samples: here, conditions), large‐*m* (features: here, gene expressions) datasets using only the first dimension of embedding (Cannistraci *et al*., 2010). The fact that our datasets have *n* < < *m* led us to adopt MCE algorithm for unsupervised analysis of the patterns present between the different sample conditions.

#### Clustering of genes

Genes with similar expression patterns often serve overlapping functions. Accordingly, the optimal number of clusters in the dataset was determined by performing a cluster analysis of gene expression patterns using the R package NbClust. Selected lists of expression profiles of DE genes were compiled for each hypothesis tested and clustered using the Ward's methodology.

#### GO and pathway enrichment analyses of DEG

Genes involved in common biological processes or pathways tend to show overlapping expression profiles. In gene expression profiling analyses, significantly enriched GO terms and pathways were identified by mapping all DEG to terms in the GO and KEGG databases by applying two‐sided Fisher's exact and χ^2^ tests respectively (da Huang *et al*., 2009a, 2009b). *P*‐values were corrected by calculating the FDR, and only GO and pathway terms with a FDR < 0.01 were chosen.

#### Identification of key BMM TFs

Promoter sequences of all DEG were retrieved using the RSAT and were further input into the RSAT matrix‐scan tool along with mouse‐related JASPAR matrices for TFBS prediction (Thomas‐Chollier *et al*., 2008; 2011; Turatsinze *et al*., 2008). The RSAT output was filtered using an adjusted *P*‐value < 0.05 as a cut‐off, and lists of the most significant TFBSs and their known corresponding TFs were compiled. The expression profiles of the mouse DEG were clustered and each cluster was correlated with TFs profiles using Pearson correlation in R. Finally, the clusters were annotated using GO to fully elucidate the molecular processes in which each TF was involved.

#### Screening for strain‐specific UPEC gene expression patterns

Flagella‐related gene lists were compiled based on Macnab's review on bacterial flagellar assembly (Macnab, 2003). A list of 35 genes related to the flagellar apparatus was used as background for screening our UPEC datasets. For OxyR regulon and hydrogen peroxide‐induced genes, gene lists were similarly compiled using previously published data on the response of bacteria to hydrogen peroxide (Zheng *et al*., 2001). Finally, lists of genes involved in Psp regulation were compiled, and our datasets were screened for their expression patterns. The expression patterns of the screened genes were further clustered as described above, and all results were visualized using pheatmap package in R.

#### Identification of UPEC genes associated with intramacrophage survival

Putative bacterial genes associated with intramacrophage survival were considered as those that remained highly up‐regulated at 24 hpi. Bacterial genes up‐regulated at this time point were compiled and filtered based on their significance of expression. All DEG were filtered using an FDR‐adjusted *P*‐value of the test statistic < 0.01 and a log_2_ fold change > 3. The filtered lists of DEG enabled us to screen for survival genes, cluster their expression and further annotate them using the GO database to better understand the biological processes they regulated.

### TIER VI: gene validation

#### cDNA synthesis, primer design and RT‐qPCR

cDNA was synthesized using a SuperScript III First‐Strand Synthesis kit (Invitrogen). Quantitative polymerase chain reaction was then performed on a 7900HT Fast Real‐Time PCR system (Applied Biosystems) using RNA samples from two independent biological replicates, similar to those employed for the RNA‐Seq experiments. TaqMan Fast Universal PCR Master Mix 2X (Applied Biosystems) was used for BMM gene validation, and SYBR Green Master Mix (Applied Biosystems) was used for UPEC gene validation. Each cDNA was analysed in triplicate, after which the average threshold cycle (Ct) per sample was calculated. Raw data were processed with qBase Plus software (Biogazelle), which performs downstream processing of qPCR data. The geNorm algorithm, integrated in the qBase Plus package, was used for determining the optimal number and identity of reference genes needed to normalize the data in both BMM (Actb and Polr2a) and UPEC (gapA and purC) qPCR libraries. Relative expression levels were calculated with the 2‐ΔΔCt method (ΔCt is the difference in Ct between the reference genes and the target gene products); the average Ct value for all genes was used to correct for differences in cDNA input. Other statistical procedures were performed with the R program. All steps, from the experimental design to bioinformatic analysis and gene validation, are summarized in Supporting Information Fig. S3.

#### Construction of UTI89pspA deletion mutant

Chromosomal DNA purification, PCR and DNA sequencing of PCR products was performed as previously described (Allsopp *et al*., 2010). The *pspA* gene was mutated in UTI89 using the λ‐red‐mediated homologous recombination method with some modifications (Datsenko and Wanner, 2000; Allsopp *et al*., 2012). Briefly, a three‐step PCR procedure was employed to generate a DNA fragment comprising the chloramphenicol cassette from plasmid pKD3 and two 500‐bp fragments homologous to the flanking regions of the *pspA* gene. The following primers were used: 5376_UTI89pspA FwUP (5′‐gccgtagcgagttcatca) and 5377_UTI89pspA RvUP (5′‐ggaataggaactaaggaggaagcgttgatgttggcatt), 5378_UTI89pspA Fwdn (5′‐cctacacaatcgctcaagacgccgaactgaaagccgat) and 5379_UTI89pspA Rvdn (5′‐taaacagcgccagaccga) to generate the 500bp homology arms; 3746‐Cm.3a (5′‐tcctccttagttcctattcc) and 3747‐Cm.4a (5′‐gtcttgagcgattgtgtagg) to generate the chloramphenicol resistance gene fragment. This DNA fusion product was electroporated into UTI89 harbouring plasmid pKD46, and chloramphenicol resistant mutants were selected and confirmed by PCR (using primers 5375_UTI89pspA FwSc: 5′‐tcgtcgcgcataccaacc and 5380_UTI89pspA Rvsc: 5′‐acttcatccagcaattcgc). The UTI89*pspA* mutant was confirmed by sequencing.

## Supporting information


**Fig. S1.** Intramacrophage survival of UTI89 versus 83972. Bacterial loads of UTI89 and 83972 within BMM at 2, 4 and 24 hpi in gentamicin exclusion assays were assessed by colony counting. These samples were used for RNA‐Seq analyses. Data represent average CFU ml^−1^ ± range (*n* = 2 independent experiments).

**Fig. S2.** Enriched mouse and UPEC GO terms during the course of infection. Gene ontology terms enriched in BMMs (A) and UPEC (B) during the 24 h course of infection. Each coloured square in the matrix represents a significant fold‐enrichment (log_2_) of the respective GO term at each point. Red, up‐regulated DEG; green, down‐regulated DEG.

**Fig. S3.** Bioinformatic analysis pipeline. Summary of the steps followed for the generation and analysis of the RNA‐Seq data produced by next‐generation sequencing. Steps are grouped into six tiers, and details are provided on the algorithms, databases and software used for each of the analyses.

**Table S1.** Quality control of RNA‐Seq libraries.

**Table S2.** Alignment statistics of RNA‐Seq reads.
